# Editorial: Rising stars in aquatic microbiology: 2022

**DOI:** 10.3389/fmicb.2023.1265720

**Published:** 2023-08-15

**Authors:** Tony Gutierrez, Hongbin Liu

**Affiliations:** ^1^Institute of Mechanical Process and Energy Engineering (IMPEE), School of Engineering and Physical Sciences, Heriot-Watt University, Edinburgh, United Kingdom; ^2^Department of Ocean Science, Hong Kong University of Science and Technology, Kowloon, Hong Kong SAR, China

**Keywords:** environmental microbiology, microbial diversity, microbial ecology, ecosystem function analysis, metagenomics

In this Research Topic, we recognize the work of future leaders in the field of Aquatic Microbiology by presenting a collection of articles that showcases the high-quality work of “rising stars” that are in the early stages of their careers and on a trajectory to potentially becoming internationally recognized leaders in their field. We aim to highlight research by these upcoming leading scientists of the future across the entire breadth of Aquatic Microbiology, with advances in theory, experimental design, and methodology with applications for compelling problems. We trust that this Research Topic will give a hint of who to follow in the coming years.

In this Research Topic, five articles highlight new findings on the functions and seasonal and ecosystem effects of microbial communities in aquatic systems. To begin, the article by Chen et al. investigates the seasonal patterns, ecological function, and assembly processes of bacterioplankton communities in the Danjiangkou Reservoir (DJR), China. Using high-throughput sequencing, PICRUSt2, and other methods, this study reveals the composition, function, interaction, and assembly of bacterioplankton communities in the DJR, providing a reference for the protection of water quality and the ecological functions of DJR bacterioplankton. In the article by Summers et al., the authors assess how different stone materials that are used in seawall construction (ranging from aluminosilicates to limestone and concrete) affect biofilm formation in tropical marine waters. Their results show bacterial biofilm colonization and succession are similar across different types of stone materials, suggesting that marine biofilms converged over time to a generic marine biofilm, rather than the underlying stone substrata type playing a significant role in driving community composition. The article by Brenes-Guillén et al. reports new insights into the prokaryotic diversity and community structure, in conjunction with physicochemistry along vertical gradients, during stratification and mixing periods in Lake Cote, Costa Rica, and provides an example of lake hydrodynamic impacts on microbial communities and their function in Central American lakes with implications for other shallow, upland, and oligotrophic lake systems. Zhao et al. report on the biogeographical patterns and assembly processes of the abundant and rare bacteria from river sediment at high altitudes (Lhasa River, China) and their potential association with antibiotic resistance genes (ARGs). Their results reveal that abundant bacteria shaped by deterministic processes have a high abundance of potential ARGs in the plateau river sediment. The article by Puigcorbé et al. shows how the vertical connectivity of the prokaryotic assemblages associated with particles of three different sizes at two East Antarctic polynyas with different surface productivity is linked to the magnitude of the particle export fluxes measured using thorium-234 (^234^Th) as particle tracer. Whilst the results support recent studies evidencing links between surface productivity and deep prokaryotic communities, this study provides the first evidence of sinking particles acting as vectors of microbial diversity to depth in Antarctic polynyas and highlights the direct influence of particle export in shaping the prokaryotic communities of mesopelagic waters.

A couplet of review articles (Tian et al.; Singh et al.) focus on the microbiology of wastewater treatment plants (WWTPs). The article by Tian et al. presents a critical overview of the existing knowledge of bioaerosol emissions from WWTPs, including their nature, magnitude, and size distribution, and highlights shortcomings associated with existing sampling and analysis methods. The mini-review by Singh et al. provides an appraisal of the different types and sources of coliphage and their fate and behavior in source waters and engineered drinking water treatment systems, and provides exciting future prospects for the use of coliphages in aquatic microbiology based on current scientific evidence and practical needs.

Body size is an important ecological trait, but it has been poorly explored in microbial communities. The article by Wu and Liu examines the effect of cell size on coastal eukaryotic communities across a size continuum of 0.2–3 (pico-), 3–20 (nano-), and 20–200 mm (micro-size), and their findings suggest that the cell size of microbial eukaryotes is a phylogenetically conserved trait which is tightly associated with biogeographic patterns.

In the study by Xia et al., comparative genomics was used to reveal a new strain of flavobacteria, *Tamlana* sp. S12, which can potentially degrade complex algal polysaccharides, thus expanding our knowledge on how marine *Flavobacteriaceae* adapt to marine algal polysaccharide environments.

In summary, these articles provide a snapshot of what is to come with respect to more high-quality work from these “rising stars” across the wide breadth of Aquatic Microbiology. We hope that this Research Topic will also inspire the younger “clan” of early career researchers to what may be possible in uncovering more about our enigmatic and incredibly profound world of microbes.

## Author contributions

TG: Conceptualization, Funding acquisition, Validation, Writing—original draft, Writing—review and editing. HL: Validation, Writing—review and editing.

